# Interaction of C-Terminal Truncated Human αA-Crystallins with Target Proteins

**DOI:** 10.1371/journal.pone.0003175

**Published:** 2008-09-09

**Authors:** Anbarasu Kumarasamy, Edathara C. Abraham

**Affiliations:** Department of Biochemistry & Molecular Biology, University of Arkansas for Medical Sciences, Little Rock, Arkansas, United States of America; Mental Health Research Institute of Victoria, Australia

## Abstract

**Background:**

Significant portion of αA-crystallin in human lenses exists as C-terminal residues cleaved at residues 172, 168, and 162. Chaperone activity, determined with alcohol dehydrogenase (ADH) and βL-crystallin as target proteins, was increased in αA_1–172_ and decreased in αA_1–168_ and αA_1–162_. The purpose of this study was to show whether the absence of the C-terminal residues influences protein-protein interactions with target proteins.

**Methodology/Principal Findings:**

Our hypothesis is that the chaperone-target protein binding kinetics, otherwise termed subunit exchange rates, are expected to reflect the changes in chaperone activity. To study this, we have relied on fluorescence resonance energy transfer (FRET) utilizing amine specific and cysteine specific fluorescent probes. The subunit exchange rate (k) for ADH and αA_1–172_ was nearly the same as that of ADH and αA-wt, αA_1–168_ had lower and αA_1–162_ had the lowest k values. When βL-crystallin was used as the target protein, αA_1–172_ had slightly higher k value than αA-wt and αA_1–168_ and αA_1–162_ had lower k values. As expected from earlier studies, the chaperone activity of αA_1–172_ was slightly better than that of αA-wt, the chaperone activity of αA_1–168_ was similar to that of αA-wt and αA_1–162_ had substantially decreased chaperone activity.

**Conclusions/Significance:**

Cleavage of eleven C-terminal residues including Arg-163 and the C-terminal flexible arm significantly affects the interaction with target proteins. The predominantly hydrophilic flexible arm appears to be needed to keep the chaperone-target protein complex soluble.

## Introduction

The major proteins in the vertebrate eye lens are α-, β-, and γ-crystallins the predominant one being the α-crystallin. α-Crystallin consists of two nearly homologous subunits, namely, αA- and αB-crystallins and both having a molecular mass of 20 kDa in the monomer form and contain 173 and 175 amino acid residues respectively [1]–[3]. Both αA- and αB-crystallins belong to the class of small heat shock proteins [4] and function as molecular chaperones having the ability to prevent aggregation of partially unfolded proteins [5]–[7]. The model structure of α-crystallin consists of a globular N-terminal domain and a C-terminal domain containing an exposed C-terminal arm rich in hydrophilic amino acids, whereas the C-terminal stretch of 80–100 residues known as the ‘α-crystallin/sHsp domain’ are highly conserved [8].

C-terminal cleavage of αA-crystallin at residues 162, 168, and 172 has been reported earlier [9]–[15]. The major post-translational modification which occurs in human αA-crystallin is the loss of the C-terminal serine residue [9], [14], [15]. Enhanced cleavage of the C-terminal residue of αA-crystallin in diabetic human lenses has been reported in our earlier study, the average level of the truncated αA-crystallin increased from 30% to 50% [15]. Aziz *et al* [16] have recently reported modification in the oligomeric structure and chaperone function of the various truncated human αA-crystallins. Interestingly, the truncated αA_1–172_ exhibited significant increase in its oligomeric size as well as chaperone activity. The oligomeric size of αA_1–168_ was similar to that of αA-wild type (αA-wt) whereas the chaperone activity was moderately decreased. αA_1–162_, on the other hand, showed substantial decrease in the oligomeric size as well as the chaperone activity. If indeed chaperone to target protein binding is an essential step for αA-crystallin to operate as a molecular chaperone, characterization of the interaction of the truncated αA-crystallins with target proteins should show why their chaperone function is altered as a result of the truncation. In this study, we have used fluorescence resonance energy transfer (FRET) to study chaperone - target protein interaction using ADH and βL-crystallin, two widely used target proteins [16], [17], and recombinant αA-wt and C-terminal truncated αA-crystallins.

## Results

### Levels of fluorescence labeling of human αA-wt, C-terminal truncated αA-crystallins and the target proteins

The level of subunit exchange between two different proteins was determined by FRET. In order to determine the *in vitro* FRET level two fluorescent dyes with overlapping fluorescence spectra are necessary. SITS and LYI are widely used fluorescent dyes with spectral overlaps and safe from structural alterations due to fluorescent tags [18]–[20]. So, in the present study the amine specific fluorescent probe SITS was attached to human αA-wt and the C-terminal truncated αA-crystallins and the cysteine specific LYI fluorescent probe was attached to the target proteins ADH and βL-crystallin. The level of labeling was determined spectrophotometrically using molar extinction coefficients of 47,000 mol^−1^ cm^−1^ at 336 nm for SITS and 11,000 mol^−1^ cm^−1^ at 426 nm for LYI. The level of labeling was about 0.8, 1.12, 1.17, 1.04 and 7.1 and 1.56 moles for αA-wt, αA_1–172_, αA_1–168_, αA_1–162_, ADH and βL-crystallin respectively. Differences in the level of labeling of the two different fluorophores were taken into account while computing the data.

### Subunit exchange between labeled αA-crystallins and the target protein ADH

**Figure 1:**
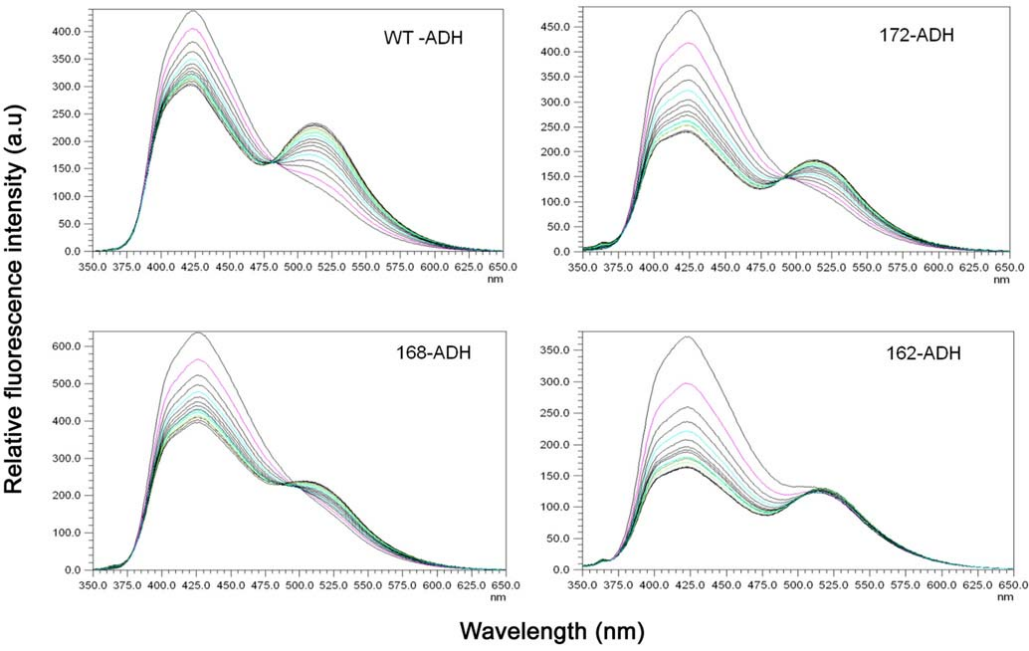
Time –dependent spectral changes in the emission spectra of SITS-labeled αA- crystallins interacting with LYI- labeled ADH. The emission spectra of fluorescence- labeled αA crystallin excited at 336 nm were recorded at every 2 min intervals at 0, 2,4,6,8,10,12,14,16,18,20,22,24,26,28 and 30 minutes after mixing of SITS – labeled αA - wt and its C-terminal truncated αA_1–172_, αA_1–168_, and αA_1–162_ with LYI – labeled ADH in 1∶1 ratio, with a final protein concentration of 1 mg/ml at 37°C. The decrease in fluorescence intensity at 426 nm of SITS- labeled αA- crystallins and concomitant increase in fluorescence intensity at 515 nm of LYI- labeled ADH is indicative of energy transfer.

**Figure 2:**
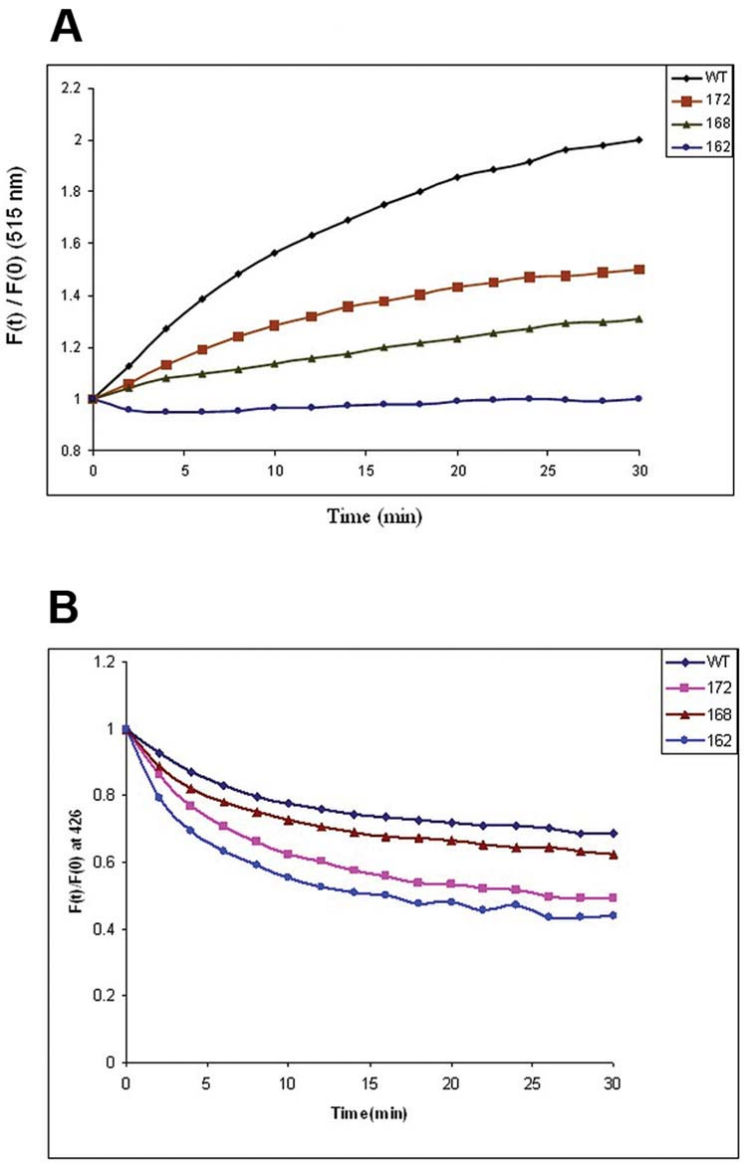
A: Relative fluorescence intensity at 515 nm due to subunit exchange of SITS – labeled αA-crystallins with LYI – labeled ADH. Time – dependent increase in acceptor fluorescence emission intensity is due to subunit exchange as evident from energy transfer from the SITS – labeled protein to the LYI – labeled protein. SITS – labeled αA wt (♦), αA_1–172_(▪), αA_1–168_ (▴) and αA_1–162_ (•) was incubated with LYI – labeled ADH. Each curve was analyzed with the best curve fit of the data to the exponential function F_t_/F_0_ = A_1_+A_2_ e^−kt^. B: Relative fluorescence intensity at 426 nm due to subunit exchange of SITS – labeled αA-crystallins with LYI – labeled ADH. Time – dependent decrease in donor fluorescence emission intensity is due to subunit exchange as evident from energy transfer from the SITS – labeled protein to the LYI – labeled protein. SITS – labeled αA wt (♦), αA_1–172_(▪), αA_1–168_ (▴) and αA_1–162_ (•) was incubated with LYI – labeled ADH. Each curve was analyzed with the best curve fit of the data to the exponential decay function F_t_/F_0_ = A_1_+A_2_ e^−kt^.

The interaction between the SITS -labeled human αA-wt and the C-terminal truncated αA-crystallins and the LYI- labeled target protein ADH was initiated by mixing equimolar concentration of the proteins in 20 mM MOPS buffer with 100 mM NaCl and 10 mM EDTA at 37°C. EDTA was used for unfolding ADH so that αA-crystallin will bind to ADH under the same condition as used for the chaperone assay. The rate of subunit exchange with ADH was determined by FRET analysis. Figure 1 shows the fluorescence spectra showing the time dependent decrease in SITS emission intensity at 426 nm and a concomitant increase in LYI fluorescence at 515 nm. After 30 min at 37°C, there was no remarkable change in the emission intensity at 515 nm due to the achievement of stable equilibrium (data not shown). The fluorescence emission spectra illustrate that the highest increase in the acceptor spectrum at 515 nm was observed in αA-wt when compared to the C-terminal truncated αA-crystallins (Fig. 1). We have calculated the rate of subunit exchange from the increase in acceptor fluorescence intensity after taking into account differences in the levels of tagging of the various proteins by the probes. Figure 2A shows the plot of Ft/ F0 of LYI at 515 nm as a function of time, where Ft and F0 are the emission intensities at time t and zero respectively. The rate constant was obtained by fitting the data to the exponential function Ft/F0 = A1+A2 e-kt, where A1 and A2 are constants and k is the rate constant for subunit exchange. The increase in the relative fluorescence intensity at 515 nm is due to fluorescence resonance energy transfer from donor SITS-labeled human αA-wt and C-terminal truncated αA-crystallins' fluorophore to the acceptor LYI-labeled ADH during the interaction. The maximum relative fluorescence intensity was seen with αA-wt whereas αA _1–172_ had lower fluorescence intensity, αA_1–168_ showed further decrease in the fluorescence intensity, and αA_1–162_ showed the lowest. The subunit exchange rates or the k values are summarized in Table 1 which is in agreement with the above observation. For instance, αA-wt had the highest k value (2.073) whereas the k values of αA_1–172_, αA_1–168_, and αA_1–162_, respectively, were 6, 24, and 43% lower. Figure 2B illustrates the decrease in the relative fluorescence at 426 nm when the fluorescence resonance energy is transferred to the acceptor target protein. The rate constant (k) values (Table 1) were higher when collected and calculated at donor energy (at 426 nm) than when calculated at acceptor energy (at 515 nm). This is believed to be was due to fluorescence quenching because no direct transfer of fluorescence energy occurs where there is no sufficient contact between the residues carrying the two probes. The amount of loss due to quenching is comparatively lower in both αA-wt and αA_1–172_ than in αA_1–168_ and αA_1–162_.

**Table 1:** Subunit exchange rate constant (k) of αA-wt and its C-terminal truncated forms interacting with ADH when increase and decrease respectively (Mean±SE).

Crystallin	Subunit exchange rate constant at 515 nm (×10^−4^ S^−1^)	Subunit exchange rate constant at 426 nm (×10^−4^ S^−1^)
αA wt+ADH	2.073±0.169	8.035±0.373
αA_1–172_+ADH	1.947±0.149	7.549±0.327
αA_1–168_+ADH	1.585±0.112	7.170±0.462
αA_1–162_+ADH	1.190±0.111	5.737±0.403

### Subunit exchange between αA-crystallins and the target protein βL-crystallin

**Figure 3:**
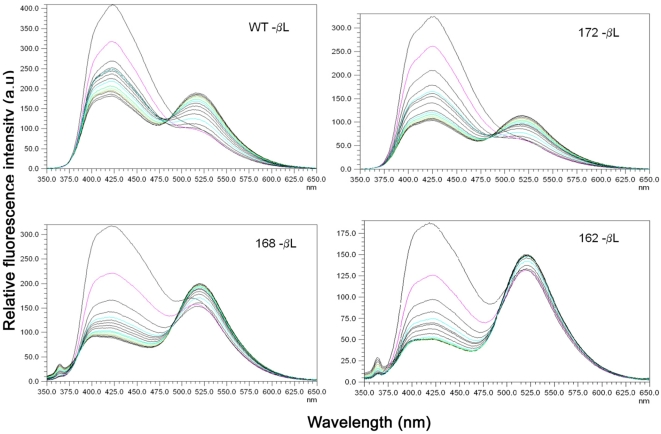
Time –dependent spectral changes in the emission spectra of the SITS-labeled αA- crystallins interacting with LYI- labeled βL- crystallin. The emission spectra of fluorescence- labeled αA crystallin excited at 336 nm were recorded at every 2 min intervals at 0, 2,4,6,8,10,12,14,16,18,20,22,24,26,28 and 30 minutes after mixing of SITS – labeled αA - wt and C-terminal truncated αA-crystallins with LYI – labeled βL- crystallin in a 1∶1 ratio, with a final protein concentration of 1 mg/ml at 62°C. The decrease in fluorescence intensity at 426 nm of SITS-labeled αA- crystallins and concomitant increase in fluorescence intensity at 515 nm of LYI- labeled ADH is indicative of energy transfer due to exchange of subunits between the two labeled proteins.

**Figure 4:**
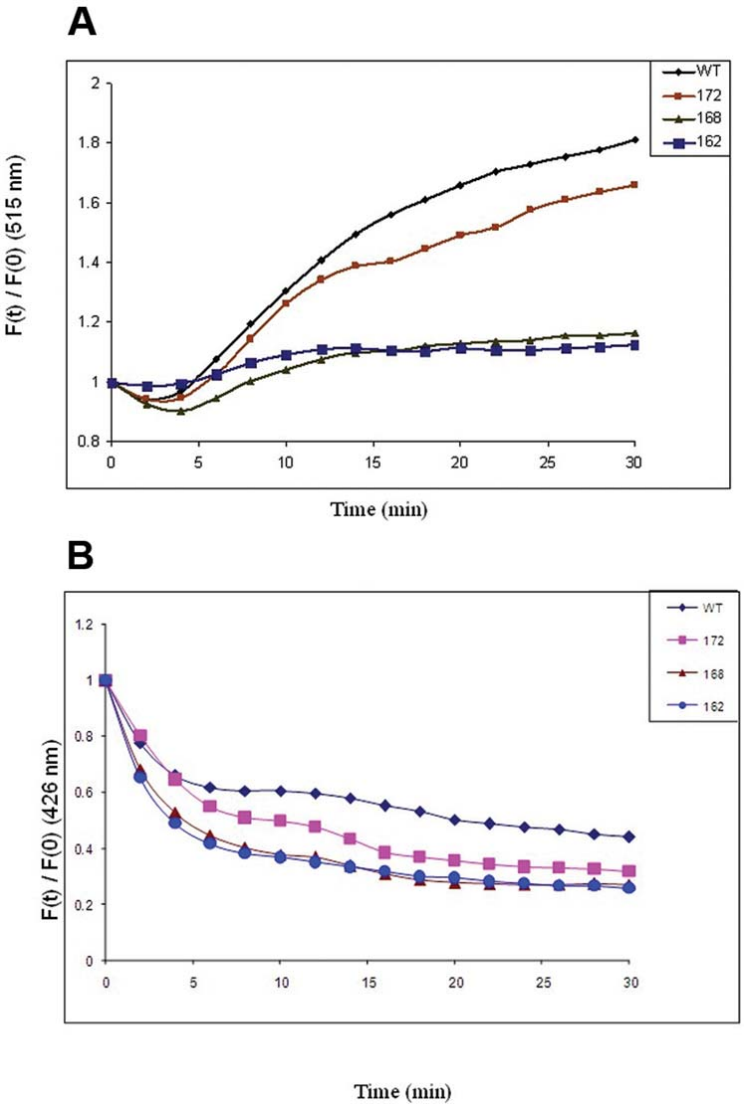
A: Relative fluorescence intensity at 515 nm of SITS – labeled αA-crystallins interacting with LYI – labeled βL- crystallin. Time – dependent increase in acceptor fluorescence in emission intensity is due to subunit exchange. Increase in the relative fluorescence intensity at 515 nm is due to energy transfer from the SITS – labeled protein to the LYI – labeled protein. SITS – labeled αA- wt (♦), αA_1–172_(▪), αA_1–168_ (▴) and αA_1–162_ (•) were incubated with LYI – labeled βL- crystallin. Each curve was analyzed with the best curve fit of the data to the exponential function F_t_/F_0_ = A_1_+A_2_ e^−kt^. B: Relative fluorescence intensity at 426 nm of SITS – labeled αA-crystallins interacting with LYI – labeled βL- crystallin. Time – dependent decrease in donor fluorescence in emission intensity is due to subunit exchange. Decrease in the relative fluorescence intensity at 426 nm is due to energy transfer from the SITS – labeled protein to the LYI – labeled protein. SITS – labeled αA- wt (♦), αA_1–172_(▪), αA_1–168_ (▴) and αA_1–162_ (•) were incubated with LYI – labeled βL- crystallin. Each curve was analyzed with the best curve fit of the data to the exponential function F_t_/F_0_ = A_1_+A_2_ e^−kt^.

The interaction between the SITS- labeled human αA-wt and the C-terminal truncated αA-crystallins and the LYI- labeled target protein βL-crystallin was initiated by mixing equimolar concentration of the proteins in 20 mM MOPS buffer with 100 mM NaCl at 62°C. Higher temperature was necessary to unfold βL-crystallin. Figure 3 shows the fluorescence spectra and Figure 4A and 4B show the increase and decrease in relative fluorescence intensity or Ft/F0 at 515 nm and 426 nm, respectively as a function of time. The k values which reflect the data in Figure 4A and 4B were summarized in Table 2. Interestingly, the acceptor gain rate constant (k) value for αA_1–172_ was maximal at 2.422 and it was about 8% higher than the value for αA-wt and about 41% higher than the values for αA_1–168_ and αA_1–162_. The same trend was noticed in donor k values determined at 426 nm also. The C-terminal truncated αA_1–172_ was maximal at 6.391 which was about 24% higher than that of αA-wt and 34% and 54% higher than those of αA_1–168_ and αA_1–162_ respectively. As mentioned above, here also at the time of energy transfer from donor fluorophore to the acceptor fluorophore some energy loss was noticed. The amount of energy lost was about 54–66%. The subunit exchange rate constant (k) values clearly showed that in αA-crystallin and the counterparts with ADH at both emission intensities (at 426 & 515 nm) the αA-wt showed the highest interaction compared to the C-terminally truncated αA-crystallins and among the C-terminally truncated αA-crystallins, αA_1–172_ showed higher k value followed by αA_1–168_ and αA_1–162_ in that order. However, the subunit exchange rate constant (k) values in αA-crystallin and counterparts with βL crystallin were higher in αA_1–172_ followed by αA-wt, αA_1–168_ and αA_1–162_. Nevertheless, the loss of energy (due to quenching) was least in αA-wt with both target proteins (ADH & βL crystallin) followed by αA_1–172_, αA_1–168_ and αA_1–162_.

**Table 2:** Subunit exchange rate constant (k) of αA-wt and its C-terminal truncated forms interacting with βL-crystallin when increase and decrease respectively (Mean±SE).

Crystallin	Subunit exchange rate constant at 515 nm (×10^−4^ S^−1^)	Subunit exchange rate constant at 426 nm (×10^−4^ S^−1^)
αA wt+βL-crystallin	2.233±0.010	4.826±0.876
αA_1–172_+βL-crystallin	2.422±0.039	6.391±0.501
αA_1–168_+βL-crystallin	1.419±0.008	4.183±0.287
αA_1–162_+βL-crystallin	1.435±0.004	3.462±0.267

### Chaperone activity of αA-wt and the C-terminal truncated αA-crystallins

**Figure 5:**
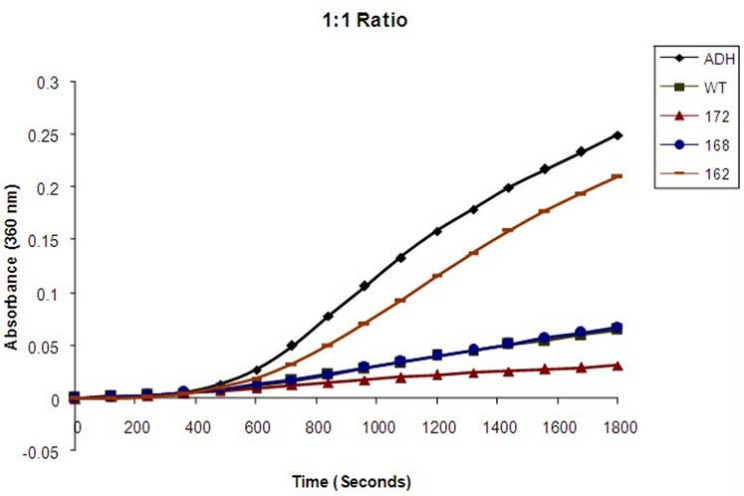
Effect of C-terminal truncation of αA-crystallin on its chaperone activity as measured with ADH as the target protein. The chaperone activity of αA-wt (▪) and C-terminal truncated αA_1–172_,(▴) αA_1–168_ (•) and αA_1–162_ (−) ADH alone (♦) determined with ADH as the target protein at 1∶1 ratio (0.14 mg/ml) denatured with EDTA at 37°C.

**Figure 6:**
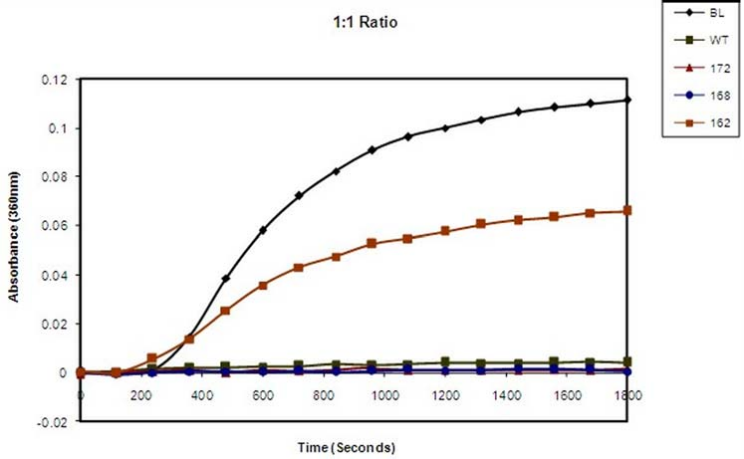
Effect of C-terminal truncation of αA-wt on its chaperone activity as measured with βL-crystallin as the target protein. The chaperone activity of αA-wt (▪) and C-terminal truncated αA_1–172_,(▴) αA_1–168_ (•) and αA_1–162_ (−) βL-crystallin alone (♦) determined with ADH as the target protein at 1∶1 ratio (0.14 mg/ml) at 62°C.

Although the chaperone activity of the truncated human αA-crystallins were reported earlier [16] the chaperone activity assay was repeated in the present study using the ratio of 1∶1 which is the same ratio as used in the FRET analysis. With ADH as the target protein, αA_1–172_ showed about 18% better chaperone activity than αA-wt, αA_1–168_ had nearly the same chaperone activity as αA-wt and αA_1–162_ showed nearly 80% loss (Fig. 5). With βL-crystallin as the protein, both αA_1–172_ and αA_1–168_ showed normal chaperone activity whereas αA_1–162_ showed nearly 60% loss in chaperone activity (Fig. 6).

## Materials and Methods

### Fluorescence probes

Lucifer yellow iodoacetamide (LYI) and 4-acetamido-4′-isothiocyanatostilbene-2,-2′-disulfonic acid (SITS) were obtained from Molecular Probes (Eugene, OR, USA). Alcohol dehydrogenase (ADH) and βL-crystallin were purchased form Sigma-Aldrich (St. Louis, USA).

### Cloning, site-directed mutagenesis, expression and purification of human αA-crystallin wild type (αA-wt), and C-terminal truncated αA-crystallins

Cloning of human αA-crystallin and subsequent subcloning into the pET-23d(+) expression vector has been described previously [21]. To generate the different C-terminally truncated human αA-crystallins lacking 1 (αA_1–172_), 5 (αA_1–168_), and 11(αA_1–162_) residues, stop codons were introduced at desired sites as described previously [16]. The pET-23d(+) expression vector harboring DNA constructs of human αA-wt and C-terminally truncated αA_1–172_, αA_1–168_, and αA_1–162_ were expressed in BL21 (DE3) PLysS *E. coli* cells. The expressed proteins were purified by Sephacryl S-300 HR size exclusion chromatography, the peak fractions collected were concentrated and repurified by molecular sieve HPLC using a 600 mm×7.8 mm BIOSEP-SEC 4000 column (Phenomenex). The purity of the protein preparations was examined by sodium dodecyl sulfate polyacrylamide gel electrophoresis (SDS-PAGE) according to Laemmli [22].

### Labeling of human αA-wt, C-terminal truncated αA-crystallins and target proteins ADH and βL-crystallin with fluorescence probes

Labeling of αA-crystallin and C-terminal truncated αA-crystallins with SITS and the target proteins ADH and βL-crystallin with LYI was done as described previously [20], [21]. Briefly, 10 fold excess of solid SITS was added to 1 ml of a protein solution (3 mg/ml) in 20 mM MOPS buffer containing 100 mM NaCl (pH 7.9) and the reaction was allowed to proceed for about 16 h at room temperature (25°C) in the dark. The unlabeled fluorescent dye was removed from the fluorescently labeled protein on a Sephadex G-25 column equilibrated with 20 mM MOPS buffer containing 100 mM NaCl (pH 7.9). Elution was performed with the same buffer, pooled and concentrated. LYI-labeled protein was prepared as described above with the same buffer in 20 fold excess of the reagent. The extent of labeling was determined spectrophotometrically using molar extinction coefficients of 47,000 mol^−1^ cm^−1^ at 336 nm for SITS and 11,000 mol^−1^ cm^−1^ at 426 nm for LYI and corrected for the contribution of the dye at 280 nm.

### Measurement of the rate of subunit exchange among αA-crystallins and the target proteins

The rate of subunit exchange was measured by the method of fluorescence resonance energy transfer (FRET). The subunit exchange reaction was initiated by mixing equal amounts (0.5 mg/ml) of SITS -labeled αA-wt and C-terminal truncated αA-crystallins (αA_1–172_, αA_1–168_ & αA_1–162_) with the same amount of LYI-labeled target proteins: 1)ADH in 20 mM MOPS buffer containing 100 mM NaCl and 10 mM EDTA (pH 7.9) at 37°C and 2) βL-crystallin in 20 mM MOPS buffer containing 100 mM NaCl (pH 7.9) at 62°C. The fluorescence emission spectra were obtained with Shimadzu RF-5301PC spectrofluorophotometer (Columbia, MD) at an excitation wavelength of 336 nm. Decrease in SITS emission intensity at 426 nm and increase in LYI emission intensity at 515 nm were recorded at every 2 min intervals and the subunit exchange rate constant (k) was calculated by curve fitting an exponential function F_t_/ F_0_ = A_1_+A_2_ e^−kt^ where A_1_ and A_2_ are constants and k is the rate constant for subunit exchange.

### Determination of Chaperone activity

The ability of αA-wt and the C-terminal truncated αA-crystallins to prevent the extent of EDTA induced aggregation of ADH and thermal aggregation of βL-crystallin was determined as described before [16], [17]. αA-wt and the C-terminal truncated αA-crystallins were mixed with equal amounts of target proteins (1∶1 ratio) at a total concentration of 0.14 mg/ml. ADH was induced to unfold with 10 mM EDTA in 50 mM PBS buffer (pH 7.9) at 37°C and the βL-crystallin unfolding was thermally induced at 62°C. The extent of aggregation was measured by monitoring the light scattering at 360 nm using Shimadzu UV 160 spectrophotometer equipped with a temperature regulated cell holder.

## Discussion

We have shown earlier that cleavage of 1, 5, and 11 C-terminal residues affects or improves chaperone activity depending on the number of residues cleaved and the chaperone assay system [16]. Chaperone activity of the truncated αA-crystallins can vary by two major factors: accessibility to the chaperone sites which are responsible for the chaperoning process or due to enhanced or decreased affinity to the substrate which is being unfolded. The present study focused on the latter aspect by studying the binding of the wild-type and the truncated αA-crystallins with the putative target proteins. The data show that the binding kinetics (k value) reflect the chaperone activity differences with the exception that αA_1–172_ showed higher chaperone activity, but, lower k value. Thus, we can conclude that the binding affinity to target proteins does not always concur with the chaperone activity data. However, it is noteworthy that all the three truncated αA-crystallins have shown decreased target protein binding.

In an earlier study, we have determined the oligomeric size, secondary and tertiary structures and chaperone activity of recombinant human αA-wt and the various C-terminal truncated αA-crystallins [16]. αA_1–172_, which is the major form of the truncated human αA-crystallin, had a molecular mass of 866 kDa as compared to 702 kDa for αA-wt, when the molecular mass was determined by dynamic light scattering. The chaperone activity of αA_1–172_ was higher than that of αA-wt, when ADH, insulin and βL-crystallin were used as target proteins and the αA-crystallin : target protein ratio varied between 1∶1 and 1∶20. In this study, we have used equal amounts of α-crystallin and target protein, ie, 1∶1 ratio, in all the assays to avoid artificially exaggerated differences between αA-wt and the truncated αA-crystallins. As shown in Figures 5 and 6, αA_1–172_ had slightly better chaperone activity than αA-wt, which confirms our previous observation [16]. However, αA-crystallin-target protein binding studies gave conflicting results. For instance, the relative fluorescence intensity due to FRET and the k values were lower in αA_1–172_ (Figs. 2 & 4; Tables 1 & 2). Both the present study and the earlier study [16] have shown αA_1–168_ having nearly the same chaperone activity as αA-wt. however, the FRET studies gave contradicting results as the relative fluorescence intensity as well as the k values were significantly lower (Figs. 2 & 4; Tables 1 & 2). With both the ADH and the βL-crystallin target protein methods, αA_1–162_ showed the lowest chaperone activity (Figs. 5 & 6) as shown earlier. Interestingly, αA_1–162_ also showed the lowest level of relative fluorescence intensity and k value during FRET studies. Thus, the cleavage of 11 C-terminal residues of αA-crystallin, which is known to affect its secondary and tertiary structures [16], severely affects its binding to target proteins which in turn affect its ability to function as a molecular chaperone.

By using FRET analysis, we have recently investigated the effect of cleavage of the C-terminal residues of human αA-crystallin on subunit exchange with αB-crystallin forming heteroaggregates [21]. The subunit exchange rate or the k value for αA_1–172_ interacting with αB-wt was decreased by 50% compared to αA-wt interacting with αB-wt. Likewise, the k value was decreased 40% when αA_1–168_ interacted with αB-wt, whereas interaction of αA_1–162_ with αB-wt showed 84% decrease in the k value. Thus, cleaving 11 residues including lysine-163 had shown the most effect. The importance of lysine-163 in maintaining the oligomeric structure of αA-crystallin has been demonstrated in a recent study [23]. In fact, in αA_1–162_ the secondary and tertiary structures were significantly altered and the molecular mass was substantially decreased [16]. Such changes affect binding to αB-crystallin and heterooligomerization [21] and also seem to affect binding to target proteins decreasing the chaperone activity.
